# Enumerating Pathways of Proton Abstraction Based on a Spatial and Electrostatic Analysis of Residues in the Catalytic Site

**DOI:** 10.1371/journal.pone.0039577

**Published:** 2012-06-20

**Authors:** Sandeep Chakraborty

**Affiliations:** Department of Biological Sciences, Tata Institute of Fundamental Research, Mumbai, India; National Institute for Medical Research, Medical Research Council, United Kingdom

## Abstract

The pathways of proton abstraction (PA), a key aspect of most catalytic reactions, is often controversial and highly debated. Ultrahigh-resolution diffraction studies, molecular dynamics, quantum mechanics and molecular mechanic simulations are often adopted to gain insights in the PA mechanisms in enzymes. These methods require expertise and effort to setup and can be computationally intensive. We present a push button methodology – Pr*oto*n *abstractio*n S*i*m*ulatio*n (PRISM) – to enumerate the possible pathways of PA in a protein with known 3D structure based on the spatial and electrostatic properties of residues in the proximity of a given nucleophilic residue. Proton movements are evaluated in the vicinity of this nucleophilic residue based on distances, potential differences, spatial channels and characteristics of the individual residues (polarity, acidic, basic, etc). Modulating these parameters eliminates their empirical nature and also might reveal pathways that originate from conformational changes. We have validated our method using serine proteases and concurred with the dichotomy in PA in Class A *β*-lactamases, both of which are hydrolases. The PA mechanism in a transferase has also been corroborated. The source code is made available at www.sanchak.com/prism.

## Introduction

Evolution has honed enzymes to efficiently and selectively catalyze biochemical reactions. Catalysis entails specific functional groups of the enzyme to be positioned appropriately with respect to the substrate [1]. Of later, the induced fit postulation has gained more acceptance over the lock and key model for catalysis [2]. The formation and rupturing of bonds after substrate binding is achieved by different modes of catalysis (metal-ion, acid-base, covalent, etc). Proton abstraction (PA) in the active site of the enzyme is a common feature in the various modes of catalysis.

The mechanism of PA often remains enigmatic despite of intense research. A classic example is the debate surrounding the base (Lys73 or Glu166) responsible for deprotonating the active site serine (Ser70) in Class A *β*-lactamases [3]–[9]. In contrast, His57 is unanimously accepted to be the base that abstracts the proton from Ser195 in serine proteases [10]–[12]. Ultrahigh-resolution diffraction studies [3], [4], [13], [14], molecular dynamics, quantum mechanics and molecular mechanic simulations [15]–[17] are methods usually applied to gain insights in the PA mechanisms in enzymes. These methods require considerable expertise for setting up the simulations and can be computationally intensive. A fast, simple and accurate method to probe the active site for possible ways of achieving the deprotonation of a known nucleophile would be quite useful for such studies.

**Figure 1:**
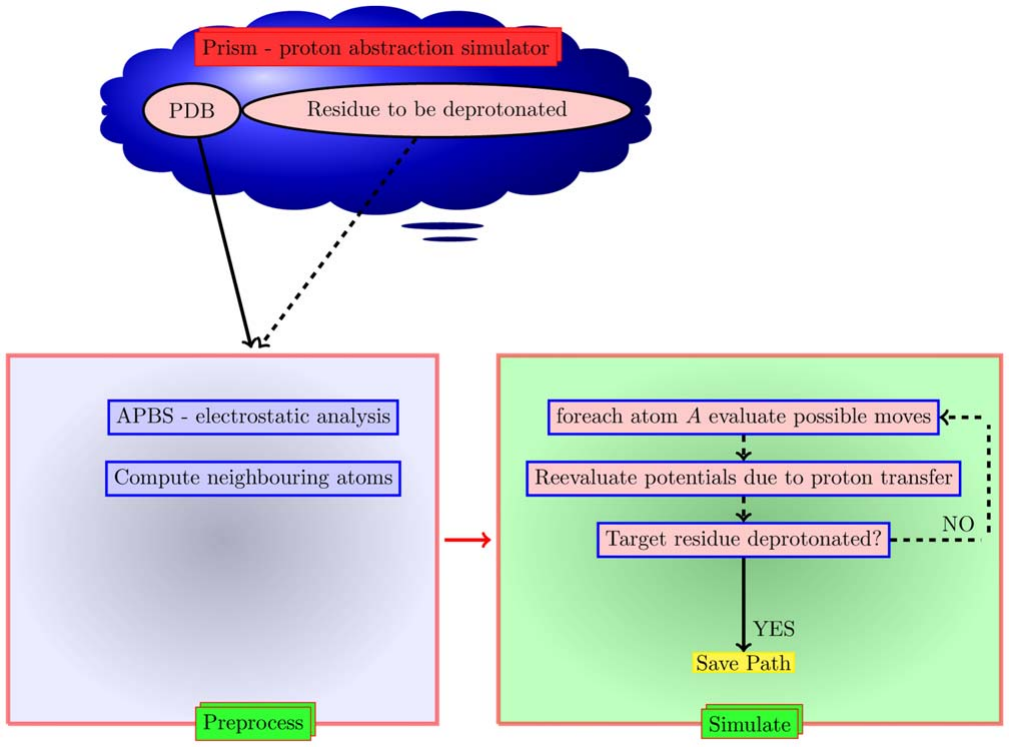
Flow for PRISM. A push button methodology for enumerating the possible pathways of proton abstraction in a protein with known 3D structure based on the spatial and electrostatic properties of residues in the proximity of a given nucleophilic residue.

We have previously established a computational method (CLASP) based on the spatial and electrostatic properties of residues for the detection of active sites and predicting unknown functions in proteins [18]. CLASP has been extended to define a generic methodology to quantify promiscuity (the catalysis of reactions distinct from the one the protein has evolved to perform, but using the same domain) in a wide range of proteins [19]. Analysis based on the potential difference between the catalytic residues in Class A *β*-lactamases identified the dichotomy in the PA mechanism and germinated the idea of a method that would enumerate the possible pathways for PA. We present an automated computational methodology – Pr*oto*n *abstractio*n S*i*m*ulatio*n (PRISM) – to enumerate the various pathways of PA based on the spatial and electrostatic properties of residues in the proximity of a known nucleophilic residue (Fig. 1).

The goal of achieving deprotonation of a nucleophilic residue can be theoretically achieved through multiple pathways. PRISM enumerates all these possibilities. Proton movements based on distances, spatial channels, potential differences and characteristics of the individual residues (polarity, acidic, basic, etc.) are iterated with simultaneous recalibration of potentials of the residues concerned. The paths that result in the desired goal of deprotonating the nucleophile are saved during these simulations, and are the final output at the end of the simulation.

PRISM has been validated using serine proteases, Class A *β*-lactamases (both hydrolases) and a serine transferase [20]. Such results demonstrate that the simplistic model of PRISM enables it be fast and simple without compromising on its ability to extract the correct proton abstraction pathways.

## Results

We present the results of running PRISM on three well known catalytic reactions involving the deprotonation of a nucleophile – Class A *β*-lactamases, serine proteases and serine hydroxymethyltransferase.

### 1. Class A *β*-lactamases

*β*-lactamases are the chief cause of bacterial resistance to penicillins, cephalosporins and related *β*-lactam compounds [21], [22]. They inactivate antibiotics by hydrolyzing the amide bond of the *β*-lactam ring yielding biologically inactive products. The Ambler classification [23], [24] has four classes – Classes A, C and D [25], [26] have a nucleophilic serine at the active site, while Class B *β*-lactamases are metallo-enzymes [27], [28].

**Figure 2:**
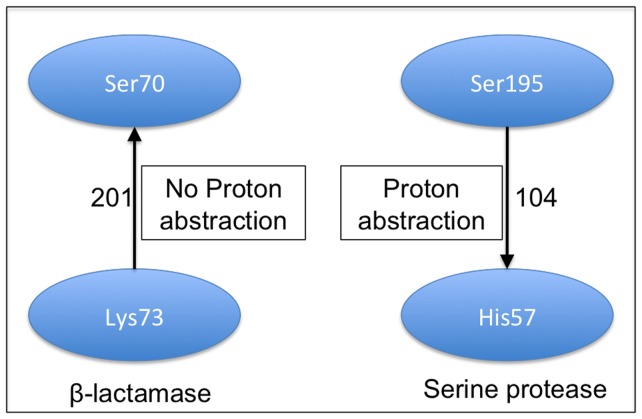
The differences in the way the nucleophilic serine is deprotonated inβ-lactamases and serine proteases. The potential differences are annotated on the edges, the direction of the edge indicating the direction of the potential difference. Ser70 cannot donate proton to Lys73 because of reverse potential gradient in β -lactamase. In serine proteases, the Ser195 however has the correct potential to donate the proton to His57.

The Class A enzymes (TEM, SHV, etc. and the newly emerging extended-spectrum *β*-lactamases) have a diverse substrate profile and are the common *β*-lactamases observed in clinical isolates. The roles of Lys73 and Glu166 in the acylation step as the catalytic base required to deprotonate the Ser70 is highly debated. The ambiguity on the role of Lys73 in deprotonating Ser70 as the sole base is evident from the reversed sign of the potential difference (PD), which suggests that Lys73 by itself cannot act as the base required to abstract the proton from Ser70 (Fig. 2).

**Figure 3:**
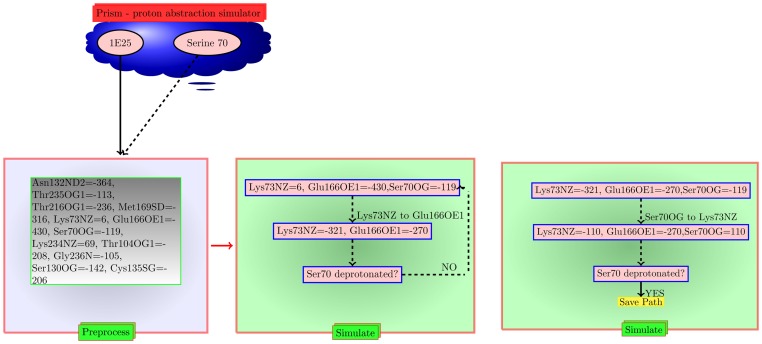
PRISM Simulation steps in PDBid:1E25. We show the simulation steps that identifies [Lys73NZ->Glu166OE1, Ser70OG->Lys73NZ] as a possible path for abstracting the proton from Ser70 in a Class A β -lactamases (PDB id: 1E25). The values associated with each atom is the potential at that atom computed using APBS.

We chose 4 structures of Class A *β*-lactamases – one apo crystal structure PER-1 (PDBid:1E25 [29]), a TEM-1 with a boronic acid transition-state analog bound (PDBid:1M40 [4]), a TEM N170G mutant with increased efficiency on ampicillin (PDBid:3JYI [30]) and a SHV-1 *β*-lactamase complexed with an inhibitor (2G2U [31]). Table 1 and Table 2 shows the pairwise distance and PD between the catalytic residues in these Class A *β*-lactamases. It is evident from these tables that these distances and PD are correlated, a result that we have previously used to predict functions in proteins [18]. The paths for PA for these proteins are shown in Table 3. Fig. 3 shows the simulation steps that identifies [Lys73NZ->Glu166OE1, Ser70OG->Lys73NZ] as a possible path for abstracting the proton from Ser70 in a Class A *β*-lactamases (PDBid:1E25). The other path for PA (Ser70OG-*>*Glu166OE1) acts through an intermediate water.

**Table 1:** Pairwise distance in Å between catalytic residues in Class A β-lactamases – Ser70(a), Lys73(b), Ser130(c), [Arg/Lys]234(d), Glu166(e).

	ab	ac	ad	ae	bc	bd	be	cd	ce	de
PDB										
1E25	2.8	3.2	4.7	5.5	3.6	5.6	5	2.9	8	10
1M40	2.7	3	4.6	5.4	3.1	5.2	5	2.8	7.7	9.8
3JYI	3.1	2.9	4.6	5	3.9	5.8	4.4	2.8	7.2	9.4
2G2U	2.8	3.5	4.4	5.4	4.3	5.3	5.1	2.6	8.4	9.6
CTX BLases										
2P74	2.7	3.1	4.5	3.9	4.3	5.7	2.9	2.8	6.6	8.1
1IYS	2.9	2.8	4.2	4.1	2.7	4.7	4.3	2.9	6.3	7.9

**Table 2:** Pairwise potential difference between catalytic residues in Class A β-lactamases – Ser70(a), Lys73(b), Ser130(c), [Arg/Lys]234(d), Glu166(e).

	ab	ac	ad	ae	bc	bd	be	cd	ce	de
PDB										
1E25	−125.6	22.4	−189.1	310.7	148.1	−63.5	436.3	−211.5	288.2	499.7
1M40	−215.3	−30.9	−241.8	230.6	184.4	−26.6	445.9	−211	261.5	472.5
3JYI	−150.5	19.2	−209	322.2	169.7	−58.5	472.7	−228.1	303.1	531.2
2G2U	−201.7	22.3	−250.3	233.7	224	−48.6	435.4	−272.7	211.4	484
CTX BLases										
2P74	191.4	−48.8	198.4	456	−240.2	7.1	264.6	247.2	504.8	257.6
1IYS	−215	−27.7	−178.1	77.7	187.3	36.9	292.6	−150.4	105.3	255.7

**Table 3:** Paths for proton abstraction as enumerated by PRISM.

PDB ids	Paths for proton transfer
**β-lactamases**	
1E25,1M40,3JYI,2G2U	1) [Ser70OG->Glu166OE1] 2) [Lys73NZ->Glu166OE1, Ser70OG->Lys73NZ]
2P74	1) [Ser70OG->Asn170ND2] 2) [Ser70OG->Glu166OE1] 3) [Ser130OG->Lys73NZ, Lys73NZ->Glu166OE1 , Ser72OG->Lys73NZ, Ser70OG->Ser72OG] 4) [Ser130OG->Lys73NZ, Ser237OG->Asn170ND2, Thr235OG1->Lys234NZ, Ser72OG->Glu166OE1, Ser70OG->Ser72OG] 5) [Ser70OG->Asn132ND2] 6) [Ser130OG->Lys73NZ, Ser237OG->Asn170ND2, Ser70OG->Ser237OG] 7) [Ser130OG->Lys73NZ, Lys73NZ->Glu166OE1, Ser70OG->Lys73NZ]
1IYS	1) [LYS73NZ->ASN170ND2, SER70OG->LYS73NZ]
**Serine proteases**	
1A0J	1) [Ser195OG->His57NE2]
1GCI	1) [Ser221OG->His64NE2]
**Serine hydroxymethyltransferase**	
1CJ0	1) [Lys229NZ->His126ND1, Thr226OG1->Lys229NZ] 2) [Thr226OG1->His228ND1]

**Figure 4:**
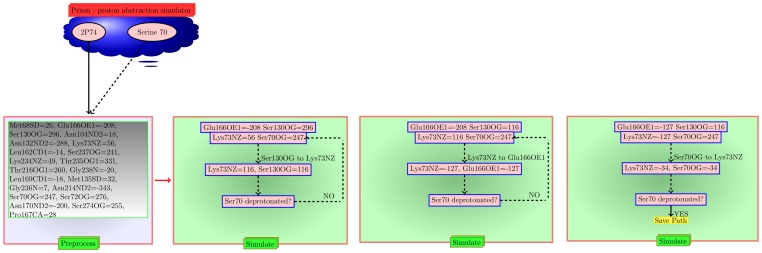
PRISM Simulation steps in PDBid:2P74. We show the simulation steps that identifies [Ser130OG->Lys73NZ, Lys73NZ->Glu166OE1, Ser70OG->Lys73NZ] as a possible path for abstracting the proton from Ser70 in a Class A β -lactamases (PDB id: 2P74). The values associated with each atom is the potential at that atom computed using APBS.

We now consider 2 CTX-M type enzymes – PDBid:2P74 [7] and PDBid:1IYS [32] – in which the Lys73 has more flexibility than other classes of Class A *β*-lactamases. Table 1 shows that these proteins differ with respect to the positioning of the OE1 atom of Glu166. This has implications with respect to PD calculations, and the deviations in PD can be seen in Table 2. For example, the distance between the functional atoms of Lys73 and Glu166 in the PDBid:1E25 and PDBid:2P74 are 5.0Å and 2.9Å respectively, while the PD between the respective atoms are 436 units and 234 units. The paths for PA in protein PDBid:2P74 are shown in Table 3. One such path in PDBid:2P74 is [Ser130OG->Lys73NZ, Lys73NZ->Glu166OE1, Ser70OG->Lys73NZ], the simulation steps for which are shown in Fig. 4. Note that only after Ser130 donated the proton to Lys73 it was able to have the high PD required to transfer a proton to Glu166.

### 2. Serine proteases

Serine proteases cut peptide bonds in proteins using a well-known catalytic triad – (Ser195, His57, Asp102) [10]. The precise synchronized action between these residues is played out within a cleft in which the substrate fits in and is subsequently cleaved off. PRISM extracts the correct path (Table 3) for PA in a trypsin-like protease (PDBid: 1A0J [33] – [Ser195OG->His57NE2]) and a subtilisin-like protease (PDBid:1GCI [34] [Ser221OG-*>*His64NE2]).

### 3. Serine hydroxymethyltransferase (SHMT)

Since both serine protease and *β*-lactamases are hydrolases, we validated PRISM on an enzyme with a different mode of catalysis – a transferase (PDBid:1CJ0 [20]). SHMT is a critical enzyme of the one-carbon units and catalyzes the interconversion of serine and glycine (folate-linked one-carbon units are needed for DNA synthesis and repair and provide methyl groups in methylation reactions). Ser226 is conserved as either a Thr or Ser across all known SHMTs [20]. PRISM extracts two paths for PA in this protein – [Lys229NZ-*>*His126ND1, Thr226OG1-*>*Lys229NZ] and [Thr226OG1-*>*His228ND1] – given Thr226 as the nucleophilic residue that is to be deprotonated (Table 3).

## Discussion

In the sheltered confines of the active site, evolution has shaped the residues to be like a spring coiled for action, albeit at the cost of the thermal stability of the whole protein [35], [36]. It is this precise recognition of the substrate that sets the whole catalytic machinery rolling [37]. A static analysis of the active site should reveal, with some degree of certainty, the course of events that follows this nudge. In the current work we enumerate the possible ways of proton abstraction (PA) from a static analysis of the spatial and electrostatic properties of residues in the neighborhood of a known nucleophile.

PA mechanisms in proteins are studied through ultrahigh-resolution diffraction studies [3], [4], [13], [14], molecular dynamics, quantum mechanics and molecular mechanic simulations [15]–[17] using molecular dynamics programs [38], [39]. We present a methodology – Pr*oto*n *abstractio*n S*i*m*ulatio*n (PRISM) – to enumerate the various pathways of PA based on the spatial and electrostatic properties of residues in the proximity of a known nucleophilic residue (Fig. 1). Proton movements based on distances, spatial channels, potential differences and characteristics of the individual residues (polarity, acidic, basic, etc) are iterated with simultaneous recalibration of potentials of the residues concerned. We have validated our method using serine proteases, Class A *β*-lactamases and serine hydroxymethyltransferases.

There are quite a few limitations of our approach, the primary being the fact that we use a static image of the protein. The distances and potential differences over which PA is allowed are empirical, as is the method for recalibrating the potentials after a proton movement. Varying these parameters (made possible by small runtimes) in a sense simulates a dynamic movement of the protein. Hard limits can never paint a true picture of catalysis – if a PA is valid over 3Å, there is no reason why it should not be valid over 3.1Å. Conformational changes in the presence of substrate is accepted to play a key role in catalysis. Although proton transfer across multiple water molecules has been observed, currently PA in PRISM is limited over a single water molecule [40]. Also, proton abstraction through metal ions is not currently handled [41]. Finally, the method is highly dependent on the tool used for potential computation [42], and thus shares the limitation of similar approaches using Finite Difference Poisson-Boltzmann (FDPB) [43]–[45].

Intuitively, the potential environment of the active site encodes more than just the catalytic residues. Keeping this in mind, the simulations are confined to the active site only. To summarize, we present a fast, simple and accurate method for enumerating potential pathways for proton abstraction of a known nucleophilic residue in a protein with known structure (PRISM).

## Materials and Methods

We now detail the PRISM methodology shown pictorially in (Fig. 1). We describe the functions and also present the pseudocode.

### 1. PRISM(): Top level function

**Figure 5:**
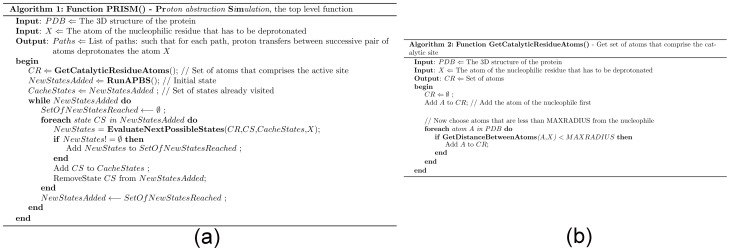
Pseudocode for PRISM. (a) Top level function. (b) Compute the set of atoms that comprise the active site.

The input to **PRISM**() (Fig. 5a) is a protein with known 3D structure, and the reactive atom of a known nucleophilic residue that has to be deprotonated (*X*). The set of atoms (*CR*) that comprises the active site (**GetActiveSiteAtoms**) is first computed and then a state of the active site is defined by the potentials of the constituent atoms. The initial state of the active site is obtained by computing the potentials of atoms in *CR* using APBS [42]. From this initial state, all possible next states based on proton transfers are iteratively computed (**EvaluateNextPossibleStates**). Proton transfers between two atoms are allowed if they are feasible from both spatial (**IsMoveSpatiallyFeasible**) and electrostatic (**IsMoveElectostaticallyFeasible**) considerations. Visited states are cached to avoid infinite looping. For each new state reached, we verify whether the target atom has been deprotonated (**IsTargetResidueDeprotonated**). If the deprotonation is achieved, then the path from the initial state to the current (goal) state is emitted as a series of proton transfers between pairs of atoms.

### 2. GetActiveSiteAtoms() Compute the set of atoms that comprise the active site

The set of atoms (*CR*) that are less than a specified distance (*MAXRADIUS*) from *X* is considered as the active site atoms (Fig 5b). Note that each residue is represented by its reactive atoms (one residue might have multiple atoms, as does histidine). *CR* includes *X* as well.

### 3. EvaluateNextPossibleStates() – Find the possible new states that can be reached by proton transfers from one initial state

**Figure 6:**
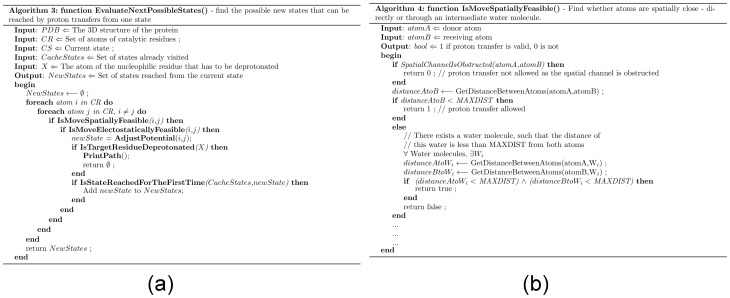
Pseudocode for PRISM. (a) Find the possible new states that can be reached by proton transfers from one state. (b) Is proton movement between to atoms spatially feasible?

This function computes the new states reachable from the current state, as defined by possible proton abstraction (PA) between each pair of atoms (Fig. 6a). The feasibility of PA is evaluated by **IsMoveSpatiallyFeasible** and **IsMoveElectostaticallyFeasible** If PA is possible, it is verified whether the last PA has achieved the goal of deprotonation of *X*. Otherwise, a new state is computed by adjusting the potentials of the two atoms involved in PA (**AdjustPotential**). The current state is tagged as visited to avoid infinite looping. Thus, each state branches into multiple new states based on the number of possible proton transfers from that state.

### 4. IsMoveSpatiallyFeasible() – Is proton movement between to atoms spatially feasible?

Proton transfer between two atoms (atomA and atomB) was regarded as possible if the distance between them is less than a specified value (*MAXDIST*), or there was a water molecule (W) such that the distance between atomA and W, and the distance between atomB and W are both less than *MAXDIST* (Fig. 6b).

Spatial hindrance from other neighboring atoms is taken into consideration. If a ball of radius 1 Å makes contact with any other atoms as it rolls from atomA to atomB, then the PA is considered as invalid between this pair of atoms.

### 5. IsMoveElectostaticallyFeasible() – Is the potential difference between the two atoms favorable for a proton transfer?

**Figure 7:**
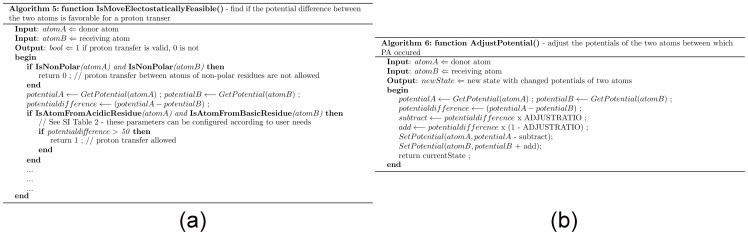
Pseudocode for PRISM. (a) Is the potential difference between the two atoms favorable for a proton transfer? (b) Adjust the potentials of the two atoms between which PA occurred.

The characteristics of the residues involved determine the potential difference required for a proton transfer (Table S1) (Fig. 7a). For example, PA is forbidden if either of the atoms belongs to a non-polar residue. Likewise PA from an acidic residue to a basic residue requires a smaller PD than a proton movement from a basic residue to an acidic residue.

### 6. AdjustPotential() – Adjust the potentials of the two atoms between which PA occured

The potentials of the two atoms are adjusted after each move, and results in a new state (Fig. 7b). The potential of the donor atom is reduced and the potential of the recipient atom is increased – as happens on a proton transfer – and the potential difference between them is reduced.

Some of the functions are self-explanatory (**GetDistanceBetweenAtoms, IsNonPolar, IsAtomFromBasicResidue, IsAtomFromAcidicResidue, IsTargetResidueDeprotonated, RunAPBS**). Table S2 shows the description of the parameters used and their default values.

### 7. Tools

Adaptive Poisson-Boltzmann Solver (APBS) and PDB2PQR packages were used to calculate the potential difference between the reactive atoms of the corresponding proteins [42], [46]. The APBS parameters are set as follows -solute dielectric: 2, solvent dielectric: 78, solvent probe radius: 1.4 Å, temperature: 298 K and 0 ionic strength. APBS writes out the electrostatic potential in dimensionless units of kT/e where k is Boltzmann's constant, T is the temperature in K and e is the charge of an electron. We extensively integrated and used the freely available BioPerl [47] modules and Emboss [48] tools.

## Supporting Information

Table S1 **Parameters used in PRISM, and their default values.**
(PDF)

Table S2 **Potential difference threshold for proton transfer.**
(PDF)
