# Supplementary material for: Enumerating Pathways of Proton Abstraction Based on a Spatial and Electrostatic Analysis of Residues in the Catalytic Site
Source: PLoS One. 2012 Jun 20;7(6):e39577. doi: 10.1371/journal.pone.0039577 (PMC3379984; doi:10.1371/journal.pone.0039577)
Supplement: Table S1 — Parameters used in PRISM, and their default values. (PDF) [file pone.0039577.s001.pdf]

Supplementary Table. 1: Potential difference thresholds for proton transfer. Potential differences are in units of kT/e where k is Boltzmann's constant, T is the temperature in K and e is the charge of an electron.

| Amino acid types in proton transfer | Potential difference |
|---|---|
| Acid->Base | 50 |
| Base->Acid | 250 |
| Acid->Acid | 150 |
| Base->Base | 130 |
| Other->Base | 100 |
| Other->Amide | 400 |
| Amide->Other | 250 |
