# Supplementary material for: Enumerating Pathways of Proton Abstraction Based on a Spatial and Electrostatic Analysis of Residues in the Catalytic Site
Source: PLoS One. 2012 Jun 20;7(6):e39577. doi: 10.1371/journal.pone.0039577 (PMC3379984; doi:10.1371/journal.pone.0039577)
Supplement: Table S2 — Potential difference threshold for proton transfer. (PDF) [file pone.0039577.s002.pdf]

Supplementary Table. 2: Parameters used in PRISM and their default values

Name	Description	Default value
MAXRADIUS	size of the active site	10 Å
MAXDIST	distance over with PA is allowed between two atoms	5 Å
BALLRADIUS	radius of the ball which is between two atoms to ascertain spatial hindrance.	1 Å
ADJUSTRATIO	ratio in which the potential difference is distributed between the atoms involved in PA	3/4
